# iProm-phage: A two-layer model to identify phage promoters and their types using a convolutional neural network

**DOI:** 10.3389/fmicb.2022.1061122

**Published:** 2022-11-04

**Authors:** Muhammad Shujaat, Joe Sung Jin, Hilal Tayara, Kil To Chong

**Affiliations:** ^1^Department of Electronics and Information Engineering, Jeonbuk National University, Jeonju, South Korea; ^2^Graduate School of Integrated Energy AI, Jeonbuk National University, Jeonju, South Korea; ^3^School of International Engineering and Science, Jeonbuk National University, Jeonju, South Korea; ^4^Advances Electronics and Information Research Center, Jeonbuk National University, Jeonju, South Korea

**Keywords:** DNA promoters, convolutional neural networks, bioinformatics, computational biology, phages

## Abstract

The increased interest in phages as antibacterial agents has resulted in a rise in the number of sequenced phage genomes, necessitating the development of user-friendly bioinformatics tools for genome annotation. A promoter is a DNA sequence that is used in the annotation of phage genomes. In this study we proposed a two layer model called “iProm-phage” for the prediction and classification of phage promoters. Model first layer identify query sequence as promoter or non-promoter and if the query sequence is predicted as promoter then model second layer classify it as phage or host promoter. Furthermore, rather than using non-coding regions of the genome as a negative set, we created a more challenging negative dataset using promoter sequences. The presented approach improves discrimination while decreasing the frequency of erroneous positive predictions. For feature selection, we investigated 10 distinct feature encoding approaches and utilized them with several machine-learning algorithms and a 1-D convolutional neural network model. We discovered that the one-hot encoding approach and the CNN model outperformed based on performance metrics. Based on the results of the 5-fold cross validation, the proposed predictor has a high potential. Furthermore, to make it easier for other experimental scientists to obtain the results they require, we set up a freely accessible and user-friendly web server at http://nsclbio.jbnu.ac.kr/tools/iProm-phage/.

## Introduction

Bacteriophages, commonly referred to as phages, are viruses that infect and destroy bacteria (Salmond and Fineran, 2015). The number of sequenced phage genomes has increased exponentially in recent decades, primarily owing to their small size and ability to bacterial infections (Silva and Echeverrigaray, 2012). This richness of genomic data necessitates the development of user-friendly bioinformatics tools to aid biologists in genome analyses. Recognition of regulatory elements is the most difficult phase in phage genome analysis. Promoters are DNA sequences responsible for transcription initiation. These sequences are difficult to identify because they are composed of short, nonconserved components. However, it is essential to comprehend and describe the genetic regulatory networks of phages, which may permit the engineering of improved phages for medicinal or biotechnological applications (Guzina and Djordjevic, 2015).

Several attempts have been made to develop promoter prediction tools for bacterial genomes. The majority of these tools use computational techniques based on-10 and-35 motifs (Sierro et al., 2008; Mishra et al., 2020; Wang et al., 2020). In contrast to these promoters with typical motifs, phage genome promoters are composed of host and phage promoters with varying motifs (Sampaio et al., 2019).

Therefore, existing tools are not suitable for identifying promoters in phages. Computational tools are required to predict promoters in phages. Prediction of phage promoters has seldom been studied. The PHIRE method (Lavigne et al., 2004) systematically scans a bacteriophage genome to determine the frequency of subsequences in a sequence. All sequences are compared, which significantly increases the running time. PromoterHunter (Klucar et al., 2010) is an online tool to identify phage promoters; however, it requires additional information as input, such as weight matrices of the two promoter elements and is limited concerning the size of the input genome sequences. The PhagePromoter tool (Sampaio et al., 2019) can be used to identify promoters across the entire phage genome. It was created using machine learning (ML) methods, such as artificial neural networks or support vector machines, in conjunction with sequence characteristics (size and score of motifs, frequency of adenine and thymine, and free energy value). Additionally, PhagePromoter can distinguish host promoters from phage promoters. However, PhagePromoter has to be used in a deterministic manner with some previous experimental or predictive knowledge, such as phage family, host bacterium species, and phage type (temperature or virulence), which limits the effectiveness of PhagePromoter. DPProm (Wang et al., 2022) is a proposed convolutional neural network (CNN)-based method for predicting phage promoters and their types as phages or hosts. However, the proposed sequence-processing workflow requires a long time for a query sequence.

Significant progress has been achieved in the essential aspects of phage promoter identification, although improvements are required in different aspects. We identified the following shortcomings of prior research:

1. Most of the aforementioned studies only predicted the promoter sequence as phage or non-promoter. Classification of predicted promoter sequences as phages or hosts was rare.
2. Most studies utilized ML models to classify predicted sequences.
3. Not all studies created a user-friendly and publicly available web server, which has proven inconvenient for practical use by experimental scientists.
4. Performance analysis of different feature encoding schemes on different ML and CNN models was not performed.
5. In the previously proposed tools, the number of false positive values for promoter prediction requires further improvement.
6. Previous studies selected non-coding regions as negative dataset, that’s makes a very easy task for the classifier on other hand trained model cannot perform well on difficult test datasets.

**Figure 1:**
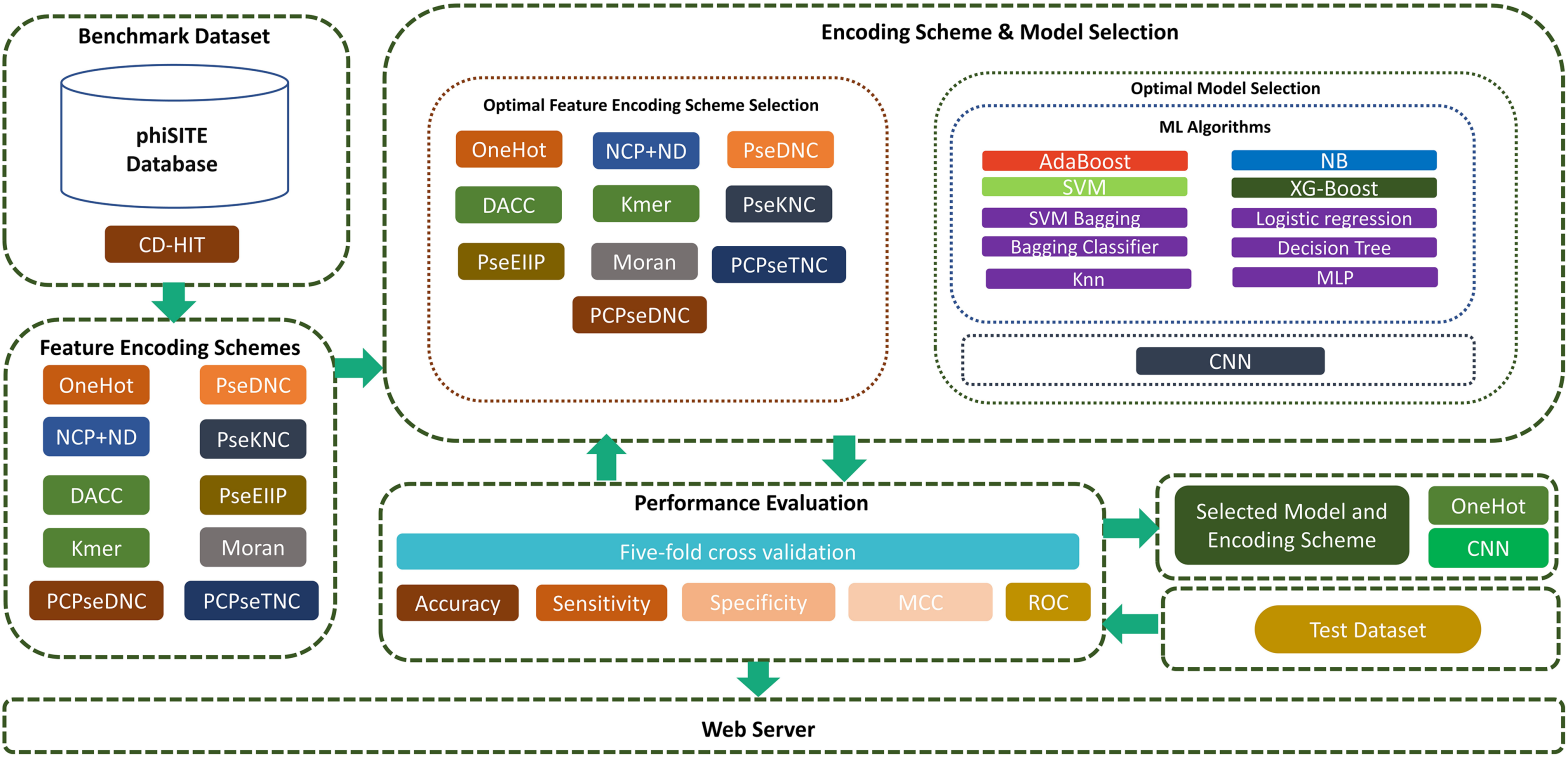
Flow diagram of iProm-phage.

In this study, we focused on overcoming these drawbacks to improve the prediction capabilities in identifying phage promoters. First, high-quality benchmark datasets were constructed. Subsequently, we extracted the best feature representation vector and model from a variety of encoding techniques, ML, and CNN models. To achieve this, we sequentially fed encoded vector sequences from all encoding methods into various ML and CNN algorithms. Based on performance evaluation, we chose the one-hot encoding technique and CNN algorithm. We investigated the sequence and properties of phage promoters and presented a two-layer model designated “iProm-phage.” In the first layer model, the query sequence is identified as a promoter or non-promoter. If it is a promoter sequence, then the second layer classifies the identified sequence as a phage promoter or host promoter. To assess model performance, we measured the accuracy (Acc), sensitivity (Sn), specificity (Sp), and Matthew’s correlation coefficient (MCC). All these parameters are frequently used in state-of-the-art methods in computational biology and bioinformatics (Rahman et al., 2019; Ali et al., 2020; Shujaat et al., 2020; Rehman et al., 2021). In addition, we evaluated the model using five-fold cross validation and receiver operating characteristic (ROC) curves. Finally, the iProm-phage web server was built in compliance with the suggested paradigm. The proposed flow diagram of the study is shown in Figure 1.

## Materials and methods

### Benchmark dataset

While developing an effective biological predictor, it is critical to select an appropriate benchmark dataset to evaluate the proposed predictive model. We prepared separate datasets for each layer of the model, as described in Sections “Dataset for the first layer” and “Dataset for the second layer.”

#### Dataset for the first layer

The promoters of phage genomes have been poorly characterized. Only the phiSITE database has identified the promoters of phage genomes (Klucar et al., 2010). The phage promoter sequence utilized in this study is the same as that used in previous studies (Sampaio et al., 2019; Wang et al., 2022). For the model’s first layer, 1,140 promoter sequences from 69 phages were collected and divided into training and test datasets; 901 promoter sequences were utilized as the training dataset and 198 promoter sequences were utilized as the test dataset. Supplementary Table S1 in Supplementary file summarize the promoter sequences from each phage genome.

The selection of a negative dataset is an important step in ensuring model performance. In previous studies, non-promoter regions were randomly selected to build a negative dataset. However, this method tends to be illogical because there is no intersection between positive and negative sets. Consequently, the model immediately detected the key differences between the two groups. Therefore, precision could not be maintained when tested on more difficult datasets. To overcome this problem, we propose a negative dataset generation technique. We created a negative dataset from positive promoter sequences by the following three steps. First, each positive sequence is divided into eight subsequences. Second, five subsequences are randomly selected and placed. Thirdly, the remaining three subsequences are placed at the same position. Using this method, each positive promoter sequence creates one negative sequence with 35–40% conserved portions from the promoter sequence. This proportion is ideal as a reliable predictor of promoter activity.

#### Dataset for the second layer

To create the positive and negative sets for the second layer of the model, promoter sequence type information as a host or phage was retrieved. The collection contains several promoters of unknown types. Finally, we collected 139 phage promoter-negative and 478 host promoter-positive samples. We randomly chose 80% of these positive and negative samples as the training dataset and 20% as the test dataset. Table 1 lists the dataset parameters for both layers.

**Table 1:** Summary of the Benchmark dataset.

Model Layer	Dataset	Promoter	Non-promoter
First layer	Training	901	901
	Test	198	198
Second layer		**Phage**	**Host**
	Training	111	382
	Test	28	96

### Methods

In this section, we briefly explain the proposed model, feature encoding techniques, and baseline models.

#### Proposed model

**Figure 2:**
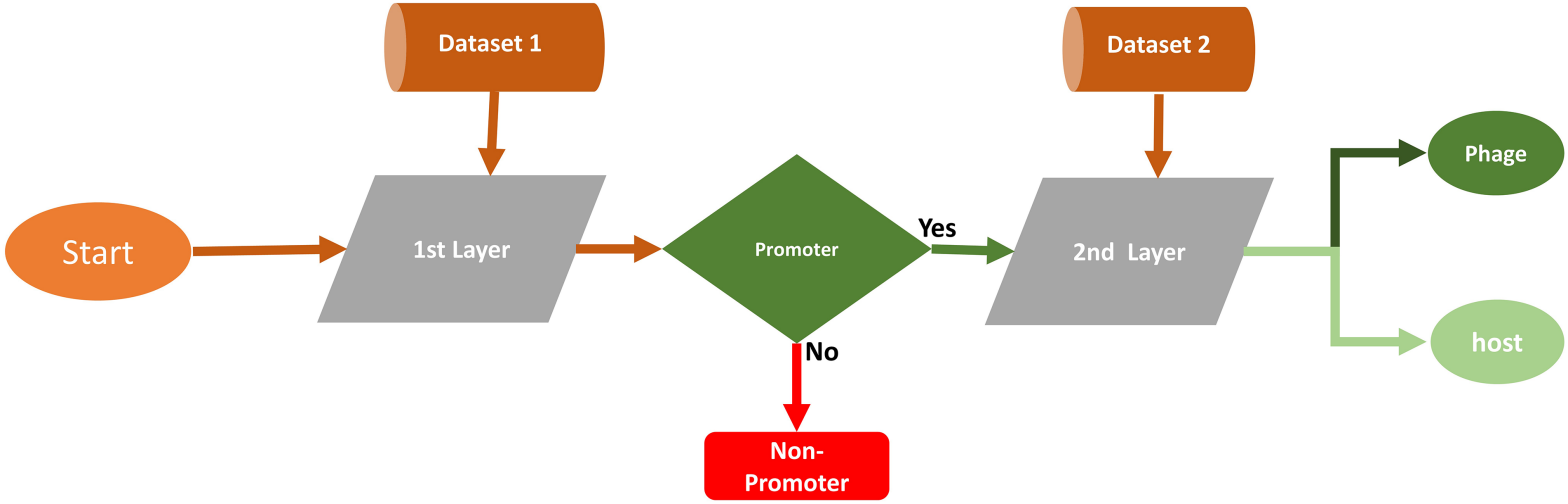
Flow diagram of the two-layer model.

The proposed two-layer model is designated “iProm-phage.” The model’s first layer predicts the query sequence as a phage promoter or non-promoter. If the predicted sequence is a phage promoter then the model’s second layer classifies it as a phage or host. Figure 2 illustrates the proposed model.

Based on performance measures, we opted for the CNN model and one-hot encoding technique for this two-layer predictor. The selection of the model and encoding technique are briefly explained in the performance measure section.

#### Convolutional neural network model architecture

The CNN is composed of 2 one-dimensional convolutional layers (Conv1D), which are followed by maximum (max) pooling and dropout layers. The filter and kernel sizes of both Conv1D is 16 and 5, respectively. The max pooling size is four with strides of two in both the max pooling layers. A dropout layer is utilized after each max pooling layer, with a value of 0.5. A flattened layer was utilized, followed by a dense layer with 64 nodes. Subsequently, we used a dropout layer with a value of 0.5. The ReLU activation function was utilized in all the Conv1D and dense layers. Finally, the dense layer is employed as an output layer with a single node and sigmoid activation function that classifies the input sequence as positive or negative based on the probability scores. The mathematical expression for the sigmoid activation function is as follows:


$$S\left(p\right)=\frac{1}{1+\exp\left(-p\right)}$$

**Figure 3:**
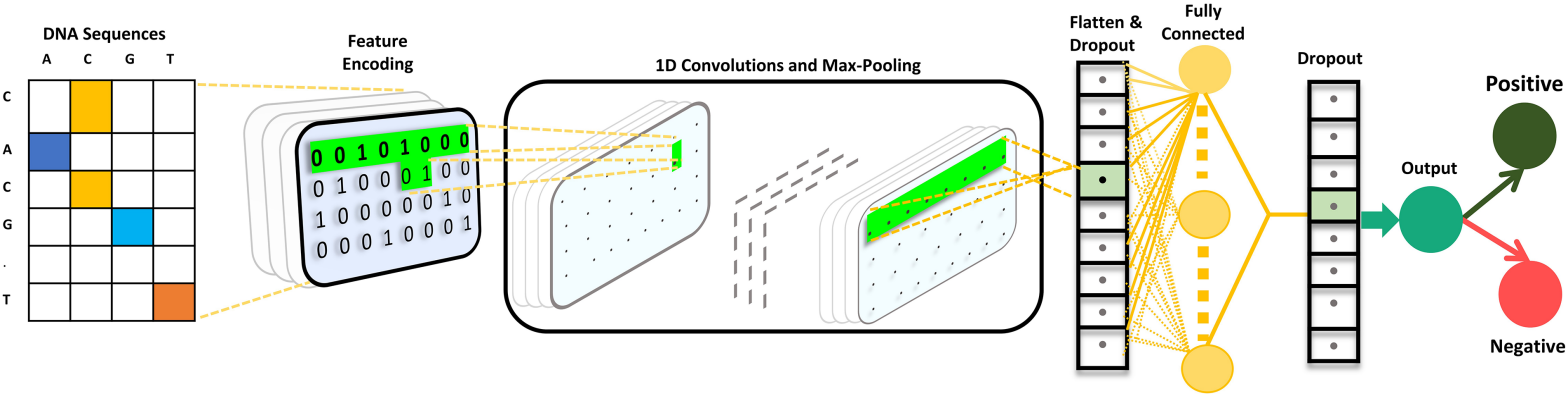
iProm-phage CNN architecture.

We used L2 regularization and bias regularization in the convolution and dense layers to ensure that the model did not overfit. The values for both regularizations were set to 0.0001. The loss function of the model is binary cross-entropy. Adam was used as the optimizer. The batch size was set to 20 with a total of 85 epochs. iProm-phage was created and trained using the Keras framework. The CNN architecture is illustrated in Figure 3.

#### Feature encoding techniques

A DNA sequence is comprised of the *A*, *C*, *G*, and *T* nucleotides. To perform computational operations, the sequence must be translated into a numerical representation. Feature encoding schemes play a vital role in creating optimal predictors. The input size should be the same for all sequences. We apply the zero-filled method to make every DNA sequence with an equal length of 99 bp. This technique was previously applied by DPProm (Wang et al., 2022). In this study, we find the best feature encoding technique among the 10 different techniques. The details of each encoding scheme are presented below.

##### One-hot feature encoding

One-hot encoding techniques are used by many state-of-the-art bioinformatics tools (Umarov and Solovyev, 2017; Liu and Li, 2019; Shujaat et al., 2021; Kim et al., 2022). Each nucleotide in a DNA sequence is represented by a four-dimensional vector, which is a vector of zeros with a single one. Nucleotide *A* is encoded as (1,0,0,0), *C* (0,1,0,0), *G* (0,0,1,0), and *T* (0, 0,0,1). Each DNA sequence can be represented by a (99,4) two-dimensional vector.

##### Nucleotide chemical property feature encoding

The chemical characteristics of the four DNA nucleic acids differ (Jeong et al., 2014). Nucleotides are classified into three types based on their chemical characteristics: hydrogen-bond strength, base type, and functional groups. Purines with two rings are represented by the letters *A* and *G*, whereas pyrimidines with one ring are represented by the letters *C* and *T*. The hydrogen bonds between *A* and *T* are weak, whereas the hydrogen bonds between *C* and *G* are strong. In terms of functional groups, the amino group includes *A* and *C*, whereas the keto group includes *G* and *T*. Each DNA sequence is represented by a three-dimensional vector (*b*, *c*, *p*) based on chemical properties, where $n_{i}$ denotes the nucleotide *n* at position *i;* hence, *b*, *c*, and, *p* were computed as follows:


$$\begin{array}{l} b_{i}\;\;=\{\begin{array}{c} 1\;\mathrm{if}\;n_{i}\in \left\{A,C\right\}\\ 0\;\mathrm{if}\;n_{i}\in \left\{G,T\right\} \end{array},\;\begin{array}{cc} \;c_{i} & = \end{array}\{\begin{array}{c} 1\;\mathrm{if}\;n_{i}\in \left\{A,G\right\}\\ 0\;\mathrm{if}\;n_{i}\in \left\{C,T\right\} \end{array},\;\\ p_{i}=\{\begin{array}{c} 1\;\mathrm{if}\;n_{i}\in \left\{A,T\right\}\\ 0\;\mathrm{if}\;n_{i}\in \left\{C,G\right\} \end{array}\; \end{array}$$

##### Dinucleotide-based auto-cross covariance feature encoding

DACC is a combination of dinucleotide-based auto-covariance (DAC) and dinucleotide-based cross covariance (DCC) encoding. DAC computes the correlation of the same physicochemical index between two dinucleotides separated by a lag distance along the sequence. DAC is calculated as:


$$\mathrm{DAC}\left(u\mathrm{,lag}\right)=\overset{L-\mathrm{lag}-1}{\underset{i=1}{\sum }}\left(\begin{array}{l} \left(P_{u}\left(R_{i}R_{i+1}\right)-{\overset{↼}{P}}_{u}\right)\\ \left(P_{u}\left(R_{i+\mathrm{lag}}R_{i+\mathrm{lag}+1}\right)-{\overset{↼}{P}}_{u}\right)/\left(L-\mathrm{lag}-1\right) \end{array}\right)$$

where u , L represent the physicochemical index and length of the sequence, respectively, and the physicochemical index u for the dinucleotide $\left(R_{i}R_{i+1}\right)$ at position i is expressed numerically as $P_{u}\left(R_{i}R_{i+1}\right)$ . ${\overset{↼}{P}}_{u}$ represents the average value of the physicochemical index u along the whole sequence, and is calculated as:


$${\overset{↼}{P}}_{u}=\overset{L-1}{\underset{j=1}{\sum }}P_{u}\left(R_{j}R_{j+1}\right)/\left(L-1\right)$$

The DAC feature vector has a dimension of N×LAG , where LAG is the maximum lag (lag = 1, 2,…, LAG) and *N* is the total number of physicochemical indices. DCC computes the correlation of two different physicochemical indices between two dinucleotides along the sequence separated by *lag* nucleic acids. Mathematically, DCC can be represented as


$$\begin{array}{l} \mathrm{DCC}\left(u_{1},u_{2}\mathrm{,lag}\right)=\overset{L-\mathrm{lag}-1}{\underset{i=1}{\sum }}\left(P_{u_{1}}\left(R_{i}R_{i+1}\right)-{\overset{↼}{P}}_{u_{1}}\right)\\ \left(P_{u_{2}}\left(R_{i+\mathrm{lag}}R_{i+\mathrm{lag}+1}\right)-{\overset{↼}{P}}_{u_{2}}\right)/\left(L-\mathrm{lag}-1\right) \end{array}$$

where $\;u_{1},u_{2}\;\mathrm{and}\;L$ represent the physicochemical indices and length of the nucleotide sequence, respectively, $P_{u_{1}}\left(R_{i}R_{i+1}\right)$ is the numerical value of the physicochemical index $u_{1}$ for the dinucleotide $\left(R_{i}R_{i+1}\right)$ at position i , and ${\overset{↼}{P}}_{ua}$ is the average value for the physicochemical index $u_{a}$ along the whole sequence, calculated as:


$${\overset{↼}{P}}_{ua}=\overset{L-1}{\underset{j=1}{\sum }}P_{ua}\left(R_{j}R_{j+1}\right)/\left(L-1\right)$$

The DCC feature vector has dimensions of N×(N−1)×LAG , where LAG is the maximum lag (lag = 1, 2,.., LAG) and *N* is the total number of physicochemical indices. Thus, the dimension of the DACC encoding is *N* × *N* × LAG, where *N* is the number of physicochemical indices and LAG is the maximum lag (lag = 1, 2, …, LAG).

##### Pseudo dinucleotide composition

PseDNC encoding incorporates both contiguous local and global sequence order information into a feature vector of the nucleotide sequence. PseDNC is mathematically defined as follows:


$$S={\left[s_{1},s_{2},\ldots ,s_{16},s_{16+1},\ldots ,s_{16+1},\ldots {\mathrm{,s}}_{16+\lambda }\right]}^{T}$$

Whereas:


$$s_{k}=\{\begin{array}{c} \frac{f_{k}}{\sum_{i=1}^{16}f_{i}+w\sum_{j=1}^{\lambda }\theta_{j}},\left(1\leq k\leq 16\right)\\ \frac{w\theta_{k-16}}{\sum_{i=1}^{16}f_{i}+w\sum_{j=1}^{\lambda }\theta_{j}},\left(17\leq k\leq 16+\lambda \right)\\ \; \end{array}$$

where $f_{k}$ (*k* = 1, 2,…, 16) is the normalized frequency of dinucleotide occurrence in the nucleotide sequence, λ represents the highest counted rank (or tie) of the correlation along the nucleotide sequence, w is the weight factor ranging from 0 to 1, and $\theta_{j}$ (*j* = 1,2,…, λ ) is the jth correlation factor and is defined as


$$\{\begin{array}{c} \theta_{1}=\frac{1}{L-2}\;\overset{L-2}{\underset{i=1}{\sum }}\theta \left(R_{i}R_{i+1,}R_{i+1}R_{i+2}\right)\\ \;\\ \theta_{1}=\frac{1}{L-2}\;\overset{L-3}{\underset{i=1}{\sum }}\theta \left(R_{i}R_{i+1,}R_{i+2}R_{i+3}\right)\\ \;\\ \theta_{1}=\frac{1}{L-2}\;\overset{L-4}{\underset{i=1}{\sum }}\theta \left(R_{i}R_{i+1,}R_{i+3}R_{i+3}\right)\;\left(\lambda <L\right)\\ \ldots \\ \;\\ \theta_{\lambda }=\frac{1}{L-1-\lambda }\;\overset{L-1-\lambda }{\underset{i=1}{\sum }}\theta \left(R_{i}R_{i+1,}R_{i+\lambda }R_{i+\lambda +1}\right)\\ \; \end{array}$$

The correlation function is given as follows:


$$\theta \left(R_{i}R_{i+1,}R_{j+1}R_{j+1}\right)=\frac{1}{\mu }\overset{\mu }{\underset{u=1}{\sum }}[P_{\mu }\left(R_{i}R_{i+1}\right)-P_{\mu }{\left(R_{j}R_{j+1}\right]}^{2}$$

where physicochemical indices are represented by *μ*, $P_{\mu }\left(R_{i}R_{i+1}\right)$ measures are the numerical values of the *u*-th (*u* = 1, 2, …, *μ*) physicochemical index of the dinucleotide $R_{i}R_{i+1}\;$ at position i and $P_{\mu }\left(R_{j}R_{j+1}\right)$ represents the corresponding value of the dinucleotide $R_{j}R_{j+1}$ at position j .Pseudo k-tupler composition (PseKNC).

PseKNC encoding uses a k-tuple nucleotide composition defined as


$$D={\left[d_{1},d_{2},\ldots ,d_{4^{k}},d_{4^{k}+1},\ldots ,d_{4^{k}+\lambda }\right]}^{T}$$

Whereas:


$$\{\begin{array}{c} \frac{f_{u}}{\sum_{i=1}^{4^{k}}f_{i}+w\sum_{j=1}^{\lambda }\theta_{j}},\left(1\leq u\leq 4\right)\\ \frac{w\theta_{u-4^{k}}}{\sum_{i=1}^{4^{k}}f_{i}+w\sum_{j=1}^{\lambda }\theta_{j}},\left(4^{k}\leq u\leq 4^{k}+\lambda \right) \end{array}$$

where λ is the total number of ranks of correlations along a nucleotide sequence, $f_{u}\left(u=1,2,\ldots ,4^{k}\right)$ is the frequency ofoligonucleotides normalized to $\overset{4^{k}}{\underset{i=1}{\sum }}f_{i}=1$ , w is the factor, and $\theta_{j}$ is defined as follows:


$$\begin{array}{l} \theta_{j}=\frac{1}{L-j-1}\overset{L-j-1}{\underset{i=1}{\sum }}\Theta \left(R_{i}R_{i+1},R_{i+j}R_{i+j+1}\right),\\ \left(j=1\mathrm{,,,;}2\mathrm{,,,;}\ldots \mathrm{,,,;}\lambda \mathrm{,,,;}\lambda <L\right) \end{array}$$

The correlation function is defined as:


$$\Theta \left(R_{i}R_{i+1},R_{i+j}R_{i+j+1}\right)=\frac{1}{\mu }\overset{\mu }{\underset{v=1}{\sum }}{\left[P_{v}\left(R_{i}R_{i+1}\right)-P_{v}\left(R_{i+j}R_{i+j+1}\right)\right]}^{2}$$

where μ represents the physicochemical index. $P_{v}\left(R_{i}R_{i+1}\right)$ is a numerical value *v*-th (*v* = 1, 2, …, *μ*). The physicochemical index of dinucleotide $\left(R_{i}R_{i+1}\right)$ at position *i* and $P_{v}\left(R_{i+j}R_{i+j+1}\right)$ represents the corresponding value of dinucleotide $\left(R_{i+j}R_{i+j+1}\right)$ at position *i* + *j*.

##### Electron-ion interaction pseudopotentials of trinucleotide

The values of nucleotides *A*, *G*, *C*, and *T* electron-ion interaction pseudopotentials (EIIP) were determined as previously described using Nair (Lavigne et al., 2004; *A*: 0.1260, *C*: 0.1340, *G*: 0.0806, *T*: 0.1335). Nucleotides in the DNA sequence are directly represented by EIIP using the EIIP value. EIIPA, EIIPT, EIIPG, and EIIPC represent the EIIP values of nucleotides *A*, *T*, *G*, and *C*, respectively, in PseEIIP encoding. A feature vector is created using the mean EIIP value of the trinucleotides in each sample, as follows:


$$V=\left[EIIP_{AAA}\cdot f_{AAA},EIIP_{AAC}\cdot f_{AAC},\ldots ,EIIP_{TTT}\cdot f_{TTT}\right]$$

##### Parallel correlation pseudo dinucleotide composition

Similar to PseDNC, PCPseDNC encoding differs in that it uses 38 default physiochemical indices for DNA instead of the six indices used in PseDNC encoding. Supplementary Table S2 in Supplementary file presents a list of 38 physicochemical indices.

##### Parallel correlation pseudo trinucleotide composition

PCPseTNC encoding is described as:


$$S={\left[s_{1},s_{2},\ldots ,s_{64},s_{64+1},\ldots {\mathrm{,s}}_{64+\lambda }\right]}^{T}$$

Whereas:


$$s_{k}=\{\begin{array}{c} \frac{f_{k}}{\sum_{i=1}^{64}f_{i}+w\sum_{j=1}^{\lambda }\theta_{j}},\left(1\leq k\leq 64\right)\\ \frac{w\theta_{k-64}}{\sum_{i=1}^{64}f_{i}+w\sum_{j=1}^{\lambda }\theta_{j}},\left(65\leq k\leq 64+\lambda \right)\\ \; \end{array}$$

where $f_{k}$ (*k* = 1, 2,…, 64) is the normalized frequency of dinucleotide occurrence in the nucleotide sequence, λ represents the highest counted rank (or tie) of the correlation along the nucleotide sequence, *w* is the weight factor ranging from 0 to 1, and $\theta_{j}$ (*j* = 1,2,…, λ ) is the *j*th correlation factor and is defined as:


$$\{\begin{array}{c} \theta_{1}=\frac{1}{L-3}\overset{L-3}{\underset{i=1}{\sum }}\Theta \left(R_{i}R_{i+1}R_{i+2},R_{i+1}R_{i+2}R_{i+3}\right)\\ \theta_{2}=\frac{1}{L-4}\overset{L-4}{\underset{i=1}{\sum }}\Theta \left(R_{i}R_{i+1}R_{i+2},R_{i+2}R_{i+3}R_{i+4}\right)\\ \theta_{3}=\frac{1}{L-5}\overset{L-5}{\underset{i=1}{\sum }}\Theta \left(R_{i}R_{i+1}R_{i+2},R_{i+3}R_{i+4}R_{i+5}\right)\;\left(\lambda <L\right)\\ \theta_{\lambda }=\frac{1}{L-2-\lambda }\overset{L-2-\lambda }{\underset{i=1}{\sum }}\Theta \left(R_{i}R_{i+1}R_{i+2},R_{i+\lambda }R_{i+\lambda +1}R_{i+\lambda +2}\right) \end{array}$$

The correlation function is defined as:


$$\Theta \left(R_{i}R_{i+1}R_{i+2},R_{j+1}R_{j+1}R_{j+2}\right)=\frac{1}{\mu }\overset{\mu }{\underset{u=1}{\sum }}{\left[\begin{array}{l} P_{u}\left(R_{i}R_{i+1}R_{i+2}\right)-\\ P_{u}\left(R_{j}R_{j+1}R_{j+2}\right) \end{array}\right]}^{2}$$

where physicochemical indices are represented by *μ*, $P_{\mu }\left(R_{i}R_{i+1}R_{i+2}\right)$ measures are the numerical values of the *u*-th (*u* = 1, 2, …, *μ*) physicochemical index of the dinucleotide $R_{i}R_{i+1}R_{i+2}\;$ at position i and $P_{\mu }\left(R_{j}R_{j+1}R_{j+2}\right)$ represents the corresponding value of the dinucleotide $R_{j}R_{j+1}R_{j+2}$ at position j .

##### Moran correlation

The distribution of amino acid characteristics along the sequence is used to create autocorrelation descriptors (Horne, 1988; Feng and Zhang, 2000; Sokal and Thomson, 2006). The amino acid properties used here are different types of amino acid indices retrieved from the AAindex Database (Kawashima et al., 2008) available at http://www.genome.jp/dbget/aaindex.html.

##### kmer

DNA sequences are represented as the occurrence frequencies of k adjacent nucleic acids in the kmer descriptor, which has been effectively used for human gene regulatory sequence prediction. The kmer descriptor (*k* = 3) is calculated as follows:


$$f\left(t\right)=\frac{N\left(t\right)}{N},t\;\varepsilon \;\left\{AAA,AAC,AAG,\ldots ,TTT\right\}\;$$

where N(t) represents the number of kmer types (*t*) and *N* is the length of the sequence.

#### Baseline models

Selection of the optimal model is a vital step in developing a novel predictor. We have utilized different ML and CNN models and, based on performance measures, selected the best model. ML models include the Adaboost (AdB) classifier, multinomial naive Bayes, extreme gradient boosting (XGboost), gradient boosting (Gboost), logistic regression (LR), K-nearest neighbor, decision tree classifier, support vector machine (SVM), multilayer perceptron classifier, and SVM bagging. A CNN is composed of two convolution layers. We used hyperparameter tuning to determine the best convolution, pooling, dropout, and dense layer parameters.

## Performance measures

In this section, we explain the evolution metrics, selection of the best model and feature encoding scheme, model performance, and model comparison.

### Evaluation metrics

In the performance assessment matrix, we used the accuracy (Acc), sensitivity (Sn), specificity (Sp), and MCC. These parameters have been used in several cutting-edge studies. The numerical representation of an evaluation matrix is expressed using the following equations:


$$\mathrm{Acc}=\frac{\mathrm{TP}+\mathrm{TN}}{\mathrm{TP}+\mathrm{TN}+\mathrm{FP}+\mathrm{FN}}$$

$$\mathrm{Sn}=\frac{\mathrm{TP}}{\mathrm{TP}+\mathrm{FN}}$$

$$\mathrm{Sp}=\frac{\mathrm{TN}}{\mathrm{TN}+\mathrm{FP}}$$

$$\mathrm{MCC}=\frac{\mathrm{TP}*\mathrm{TN}-\mathrm{FP}*\mathrm{FN}}{\left(\mathrm{TP}+\mathrm{FP}\right)\left(\mathrm{TP}+\mathrm{FN}\right)\left(\mathrm{TN}+\mathrm{FP}\right)\left(\mathrm{TN}+\mathrm{FN}\right)}$$

The terms TP, TN, FP, and FN in the aforementioned equations represent the appropriate numbers of true positives, true negatives, false positives, and false negatives, respectively.

### Selection of best model and feature encoding

**Figure 4:**
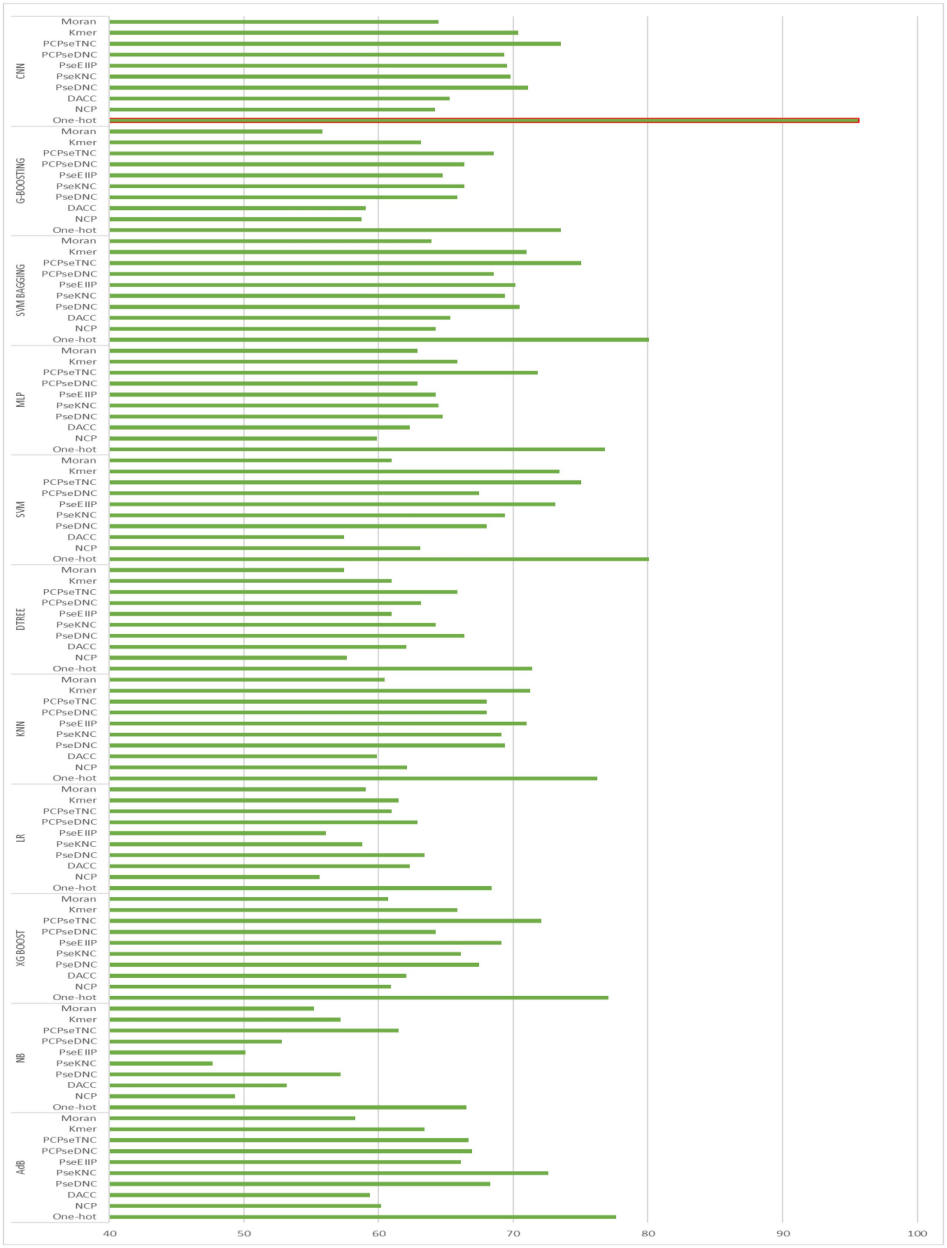
Accuracy of First layer baseline models.

**Figure 5:**
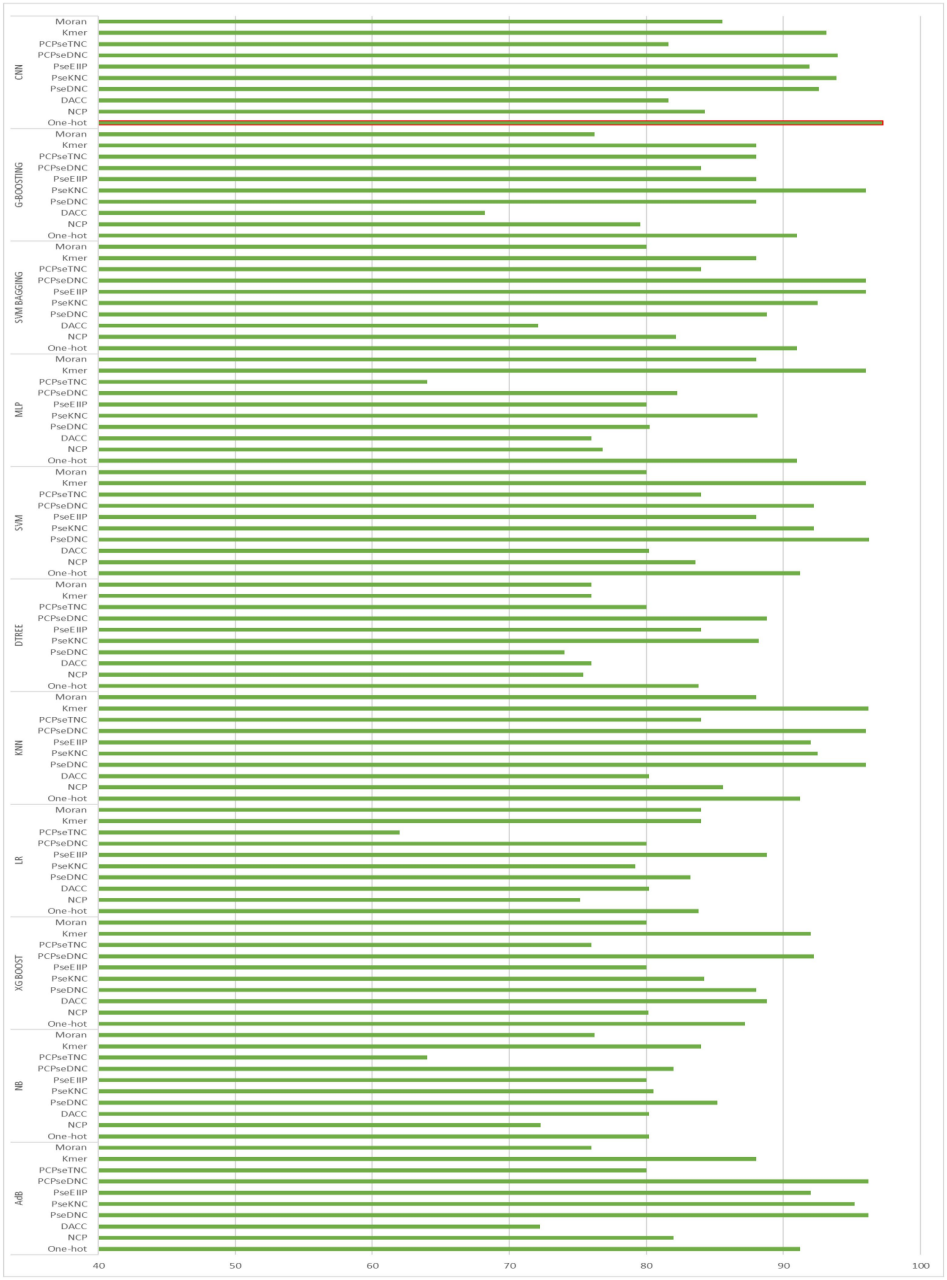
Accuracy of Second layer baseline models.

To generate an optimum model, we compared all the encoding strategies stated above to the baseline approaches. Supplementary Tables S3, S4 in Supplementary file, and Figures 4, 5 illustrate the performance of each method on various encoding schemes for the first and second layers. For the first layer of the model CNN and one-hot encoding outperformed after that AdB performed better on PseKNC feature encoding and for the second layer almost every feature encoding scheme performed good on ML and CNN algorithms, but one-hot and CNN outperformed in the second layer as well. Therefore, based on performance evaluation, we chose the CNN and one-hot encoding technique for both layers and the proposed tool “iProm-phage.”

### Model performance

**Figure 6:**
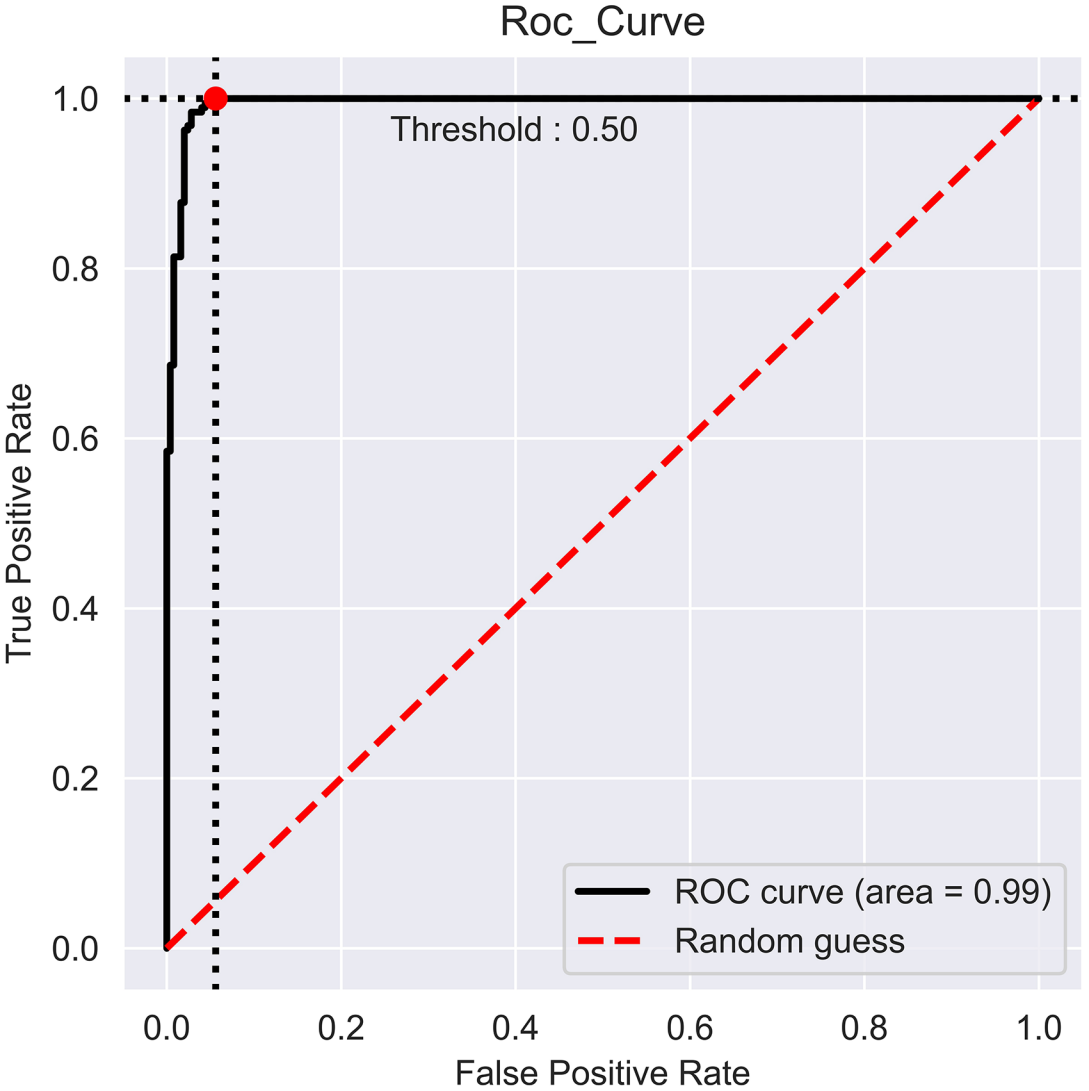
First layer ROC curve.

**Figure 7:**
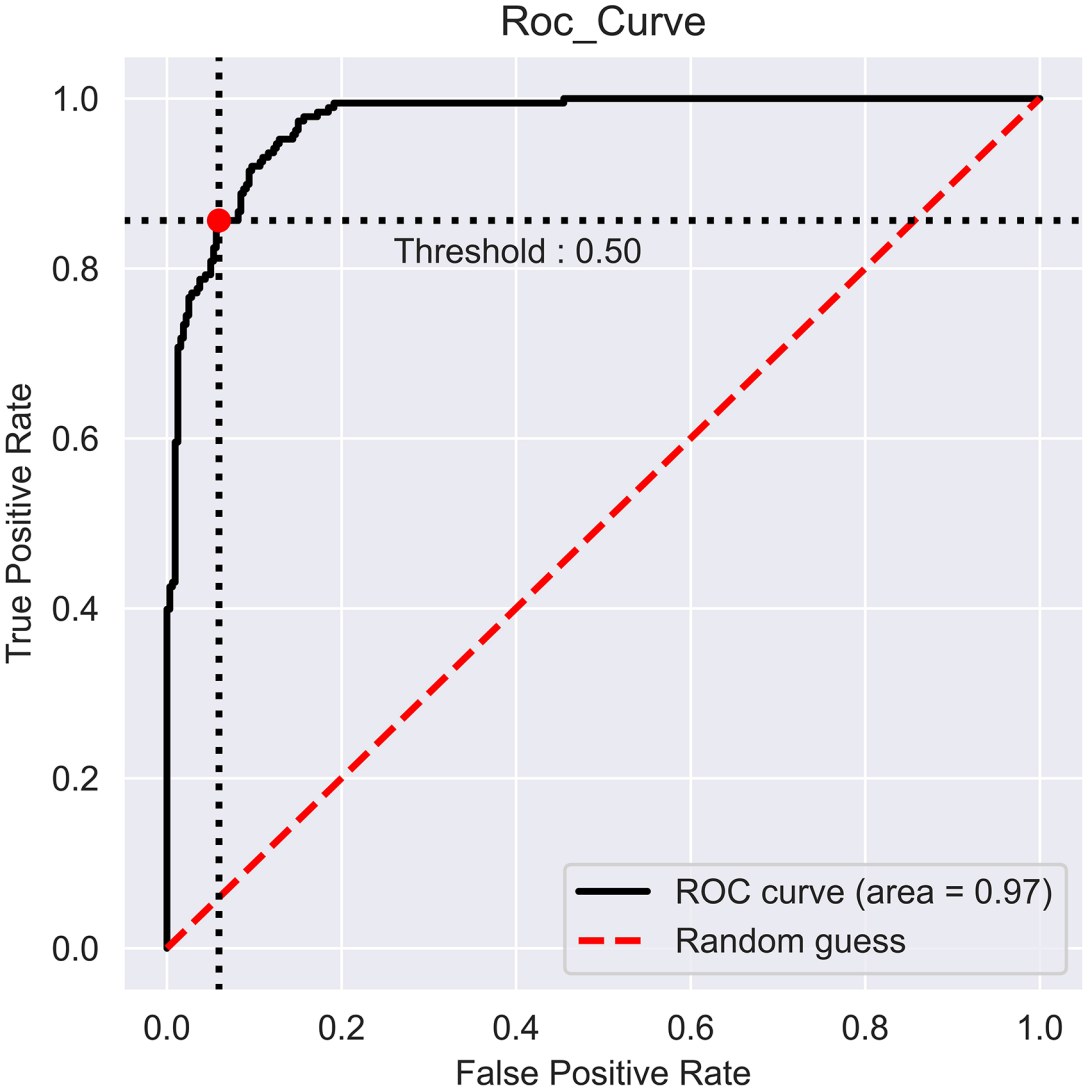
Second layer ROC curve.

The prediction performance of iProm-phage was evaluated using 5-fold cross validation. We employed the same parameters used in choosing the best model and also considered ROC curve data. The first layer of iProm-phage achieved an Acc of 95.68 93.47%, Sn of 96.12%, Sp of 92.63%, MCC of 0.872, and AUROC of 0.99 during cross validation. These findings suggest that our predictor is capable of properly recognizing whether a query sequence is a promoter. The second layer of iProm-Zea achieved values of 97.25, 94.32, 98.5%, 0.8619, and 0.97, respectively. In the test dataset model, the first layer achieved an accuracy of 94.2%, Sn 90%, Sp 90%, and MCC 0.88. The second layer obtained accuracies of 95.2%, 94.37%, 97.14%, and 0.88% for the test dataset. Figures 6, 7 depict the ROC curves for both layers of the iProm-phage model.

### Comparison with existing models

We compared iProm-phage with state-of-the-art promoter identification tools PhagePromoter and DPProm for the identification of query sequences as promoters or promoters. We measured the precision and recall for both layers to compare them with state-of-the-art methods. The following equations express precision and recall:


$$\mathrm{Recall}=\frac{\mathrm{TP}}{\mathrm{TP}+\mathrm{FN}}$$

$$\mathrm{Precison}=\frac{\mathrm{TP}}{\mathrm{TP}+\mathrm{FP}}$$

A performance comparison of the methods used for promoter identification is presented in Table 2. The superior performance of the proposed iProm-phage tool can be observed in all four performance metrics for this particular task.

**Table 2:** First layer performance comparison.

**Methods**	**Acc%**	**Precision%**	**Recall%**
PhagePromoter	92	89	87
DPProm	85.5	88.9	83
iProm-phage	95.68	94.2	93.5

We demonstrate the performance comparison between DPProm in Table 3 for promoter classification as a phage or host. The iProm-phage tool was superior to DPProm in performance for all classification tasks. The precision and recall of iProm-phage for promoter identification and classification were higher than those of DPProm, and the values were more consistent. As a result, iProm-phage showed a considerably higher score than the state-of-the-art methods in all cases.

**Table 3:** Second layer performance comparison.

**Methods**	**Acc%**	**Precision%**	**Recall%**
DPProm	93.0	95.2	96.4
iProm-phage	95.2	96.5	97.2

## Webserver

**Figure 8:**
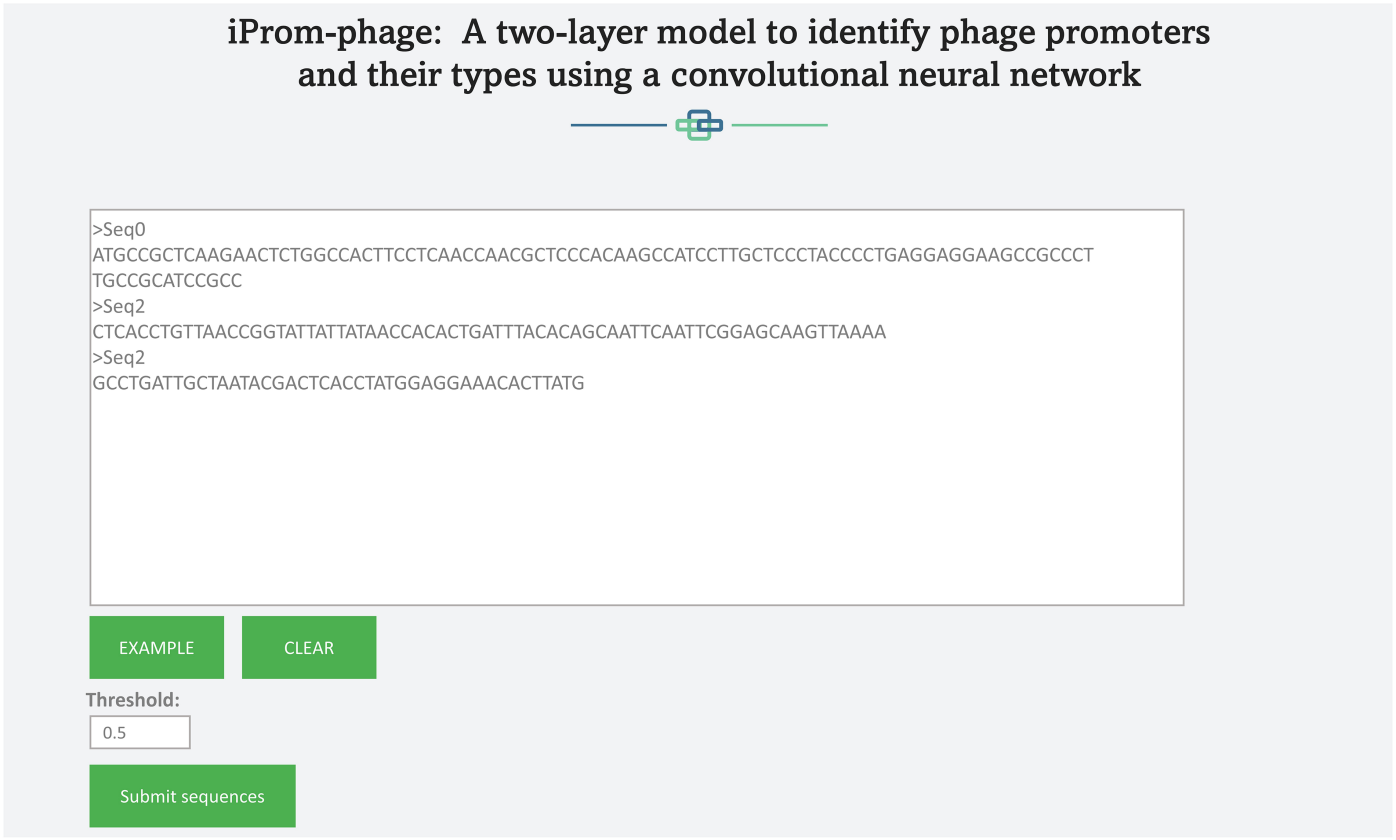
Webserver adding query sequence.

**Figure 9:**
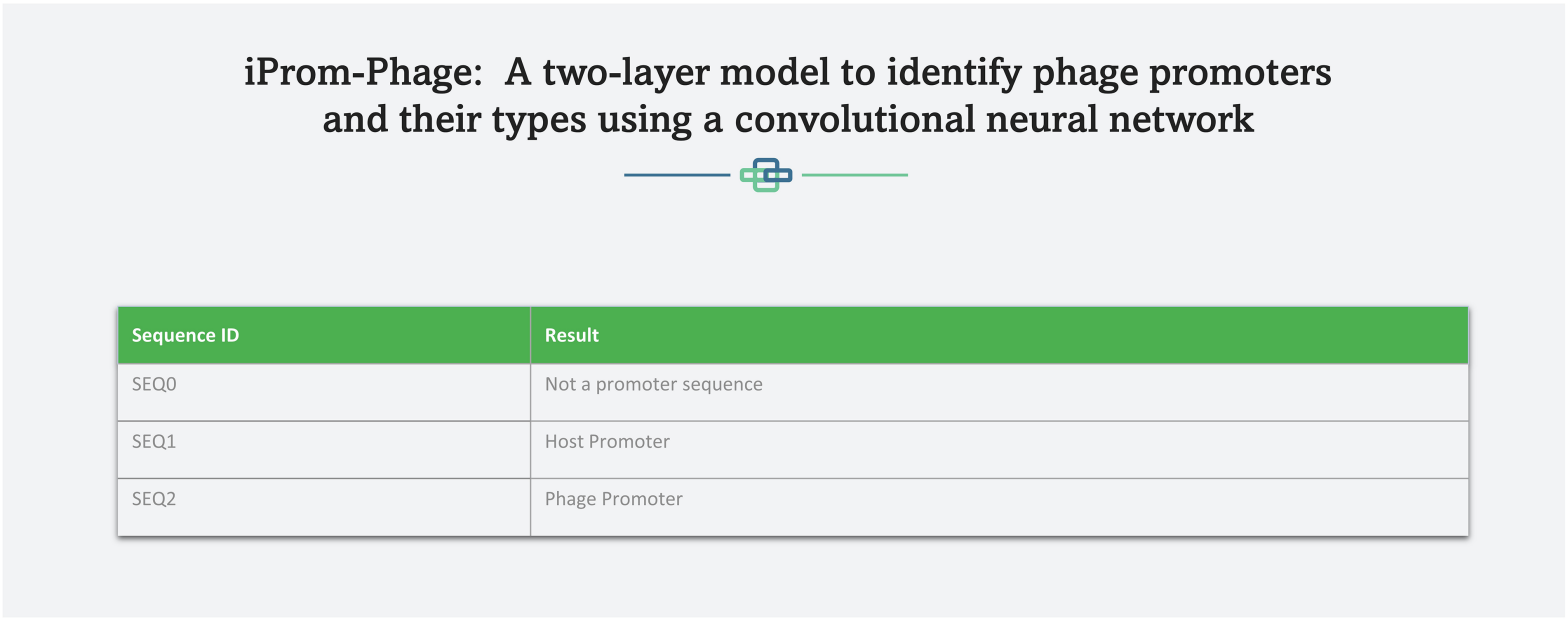
Predictor output.

A web server hosting the high performance iProm-phage tool is freely available at the following link^1^ to enable easy access to the proposed tool for the scientific community. This approach has been adopted by several scholars (Chantsalnyam et al., 2020; Ali SD et al., 2022). iProm-phage is an easy-to-use tool that can be utilized by researchers and specialists in bioinformatics. It consists of two stages first is input and second is output. To input it uses two input methods: direct sequence input and uploading a file containing sequences for prediction. Each sequence should be 99 bp long and contain the letters *A*, *C*, *G*, and *T*. Figures 8, 9 depict web server snippets; Figure 8 is an example of adding sequences for prediction and Figure 9 provides the predictor’s output. We also provide an example to better understand how to use the webserver.

## Conclusion

This work presents iProm-phage, a two-layer technique for identifying phage promoters and classifying them as phages or hosts. We developed a new method for generating negative datasets to create a robust model that performs well on tough datasets. Based on cutting-edge performance tests, we also found the best model among several ML and CNN algorithms, as well as the best feature encoding method among the 10 distinct methods. The architecture of the proposed model was evaluated using publicly available datasets. Compared to earlier techniques, the program had superior overall results. Finally, we created a web server that is available online and will be extremely useful to other experimental scientists.

## Data availability statement

The original contributions presented in the study are included in the article/Supplementary material, further inquiries can be directed to the corresponding authors.

## Author contributions

MS: conceptualization, methodology, software, writing–original draft, and writing–review and editing. JJ: methodology and writing–review and editing. HT: supervision and writing–review and editing. KC: conceptualization, validation, supervision, writing–review and editing, and funding acquisition. All authors contributed to the article and approved the submitted version.

## Funding

This work was supported in part by the National Research Foundation of Korea (NRF) grant funded by the Korean government (MSIT; nos. 2020R1A2C2005612 and 2022R1G1A1004613). This work was supported by “Human Resources Program in Energy Technology” of the Korea Institute of Energy Technology Evaluation and Planning (KETEP), granted financial resource from the Ministry of Trade, Industry & Energy, Republic of Korea (no. 20204010600470).

## Conflict of interest

The authors declare that the research was conducted in the absence of any commercial or financial relationships that could be construed as a potential conflict of interest.

## Publisher’s note

All claims expressed in this article are solely those of the authors and do not necessarily represent those of their affiliated organizations, or those of the publisher, the editors and the reviewers. Any product that may be evaluated in this article, or claim that may be made by its manufacturer, is not guaranteed or endorsed by the publisher.

## Footnotes

^1^ http://nsclbio.jbnu.ac.kr/tools/iProm-phage/
