# Supplementary material for: iProm-phage: A two-layer model to identify phage promoters and their types using a convolutional neural network
Source: Front Microbiol. 2022 Nov 4;13:1061122. doi: 10.3389/fmicb.2022.1061122 (PMC9672459; doi:10.3389/fmicb.2022.1061122)
Supplement: Supplementary file 1 [file Data_Sheet_1.docx]

Supplementary material file

iProm-phage: A two-layer model to identify phage promoters and their types using a convolutional neural network

**Muhammad Shujaat^1^, Joe Sung Jin^2,^ Hilal Tayara^3, *^ , and Kil To Chong^1,4,*^**

^1^Department of Electronics and Information Engineering, Jeonbuk National University, Jeonju 54896, Republic of Korea. ^2^ Graduate School of Integrated Energy AI, Jeonbuk National University, Jeonju 54896, Republic of Korea. ^3^ School of International Engineering and Science, Jeonbuk National University, Jeonju 54896, Republic of Korea, ^4^ Advances Electronics and Information Research Center, Jeonbuk National University, Jeonju 54896, Republic of Korea.

Contents:

Contents

[Table s1. Promoter sequences from each phage genome 2](#_Toc115789094)

[Table s2. Names of the 38 physicochemical dinucleotides indices 3](#_Toc115789095)

[Table s3. First layer performance of baseline models on different feature encoding techniques 4](#_Toc115789096)

[Table s4. Second layer performance of baseline models on different feature encoding techniques 8](#_Toc115789097)

# Table s1. Promoter sequences from each phage genome

| **List of phage genomes used in the training data** | | | |
| --- | --- | --- | --- |
| Bacillus phage B103 | Bacillus phage GA-1 | Bacillus phage Nf | Bacillus phage phi105 |
| Bacillus phage phi29 | Enterobacteria phage 933W | Enterobacteria phage HK022 | Enterobacteria phage lambda |
| Enterobacteria phage Mu | Enterobacteria phage P1 | Enterobacteria phage P2 | Enterobacteria phage P22 |
| Enterobacteria phage P4 | Enterobacteria phage SP6 | Enterobacteria phage T3 | Enterobacteria phage T4 |
| Enterobacteria phage T7 | Enterobacteria phage VT2-Sakai | Escherichia Stx1 converting phage | Pseudomonas phage gh-1 |
| Pseudomonas phage phiKMV | Salmonella phage HK620 | Staphylococcus phage P68 | Streptococcus phage Cp-1 |
| Streptomyces phage phiC31 | Stx2 converting phage I | Stx2 converting phage II | Yersinia phage phiYeO3-12 |
| Klebsiella phage KP34 | Klebsiella phage F19 | Klebsiella phage SU503 | Klebsiella phage SU552A |
| Klebsiella phage K244 | Enterobacteria phage vB_PcaM_CBB | Klebsiella phage K11 | Enterobacteria phage MmP1 |
| Yersinia phage phiA1122 | Enterobacteria phage BA14 | Cronobacter phage GAP32 | Pseudomonas phage LKD16 |
| Pseudomonas phage LKA1 | Pf-10 | PPA-ABTNL | LUZ19 |
| phiKF77 | PT2 | PT5 | Escherichia phage T5 |
| Enterobacteria phage phi80 | Enterobacteria phage 186 | Lactococcus phage asccphi28 | Enterobacteria phage ES18 |

# Table s2. Names of the 38 physicochemical dinucleotides indices

| Base stacking | Protein induced deformability | B-DNA twist | A-Phylicity | Propeller twist |
| --- | --- | --- | --- | --- |
| DNA denaturation | Bending stiffness | Protein DNA twist | Aida_BA_transition | Breslauer_dG |
| Electron interaction | Hartman_trans_free_energy | Helix-Coil_transition | Lisser_BZ_transition | Polar_interaction |
| SantaLucia_dS | Sarai_flexibility | Stability | Sugimoto_dG | Sugimoto_dH |
| Duplex tability(disruptenergy) | Stabilising energy of Z-DNA | Breslauer_dS | Ivanov_BA_transition | SantaLucia_dH |
| Watson-Crick interaction | Dinucleotide GC Content | Twist | Stacking_energy | Sugimoto_dS |
| Slide | Rise | Tilt | Roll | Shift |
| Duplex stability:(freeenergy) | Breslauer_dH | SantaLucia_dG |  |  |

# Table s3. First layer performance of baseline models on different feature encoding techniques

| **Classifier** | **Encoding Scheme** | **Sn** | **Sp** | **Acc** | **MCC** |
| --- | --- | --- | --- | --- | --- |
| AdB | One-hot | 81.96 | 75.94 | 77.62 | 0.606 |
|  | NCP | 59.23 | 61.37 | 60.20 | 0.155 |
|  | DACC | 53.80 | 65.69 | 59.34 | 0.195 |
|  | PseDNC | 65.78 | 70.94 | 68.29 | 0.367 |
|  | PseKNC | 76.96 | 68.58 | 72.62 | 0.456 |
|  | PseEIIP | 64.39 | 68.29 | 66.12 | 0.324 |
|  | PCPseDNC | 68.32 | 65.86 | 66.93 | 0.339 |
|  | PCPseTNC | 64.01 | 69.07 | 66.66 | 0.331 |
|  | Kmer | 63.68 | 63.15 | 63.41 | 0.268 |
|  | Moran | 56.97 | 59.39 | 58.26 | 0.163 |
| NB | One-hot | 77.41 | 64.71 | 66.51 | 0.38 |
|  | NCP | 57.35 | 48.53 | 49.36 | 0.025 |
|  | DACC | 52.36 | 58.21 | 53.21 | 0.16 |
|  | PseDNC | 70.21 | 55.27 | 57.18 | 0.17 |
|  | PseKNC | 66.66 | 47.05 | 47.69 | 0.13 |
|  | PseEIIP | 50.12 | 52.32 | 50.112 | 0.22 |
|  | PCPseDNC | 61.29 | 52.07 | 52.84 | 0.12 |
|  | PCPseTNC | 67.44 | 59.71 | 61.51 | 0.230 |
|  | Kmer | 72.41 | 54.34 | 57.18 | 0.194 |
|  | Moran | 58.32 | 49.32 | 55.23 | 0.172 |
| XG BOOST | One-hot | 96.86 | 72.15 | 77.08 | 0.618 |
|  | NCP | 80.30 | 55.93 | 60.94 | 0.221 |
|  | DACC | 73.58 | 60.12 | 62.05 | 0.237 |
|  | PseDNC | 89.04 | 62.16 | 67.47 | 0.401 |
|  | PseKNC | 91.86 | 58.30 | 66.12 | 0.425 |
|  | PseEIIP | 88.8 | 62.72 | 69.10 | 0.440 |
|  | PCPseDNC | 85.5 | 59.33 | 64.22 | 0.349 |
|  | PCPseTNC | 86.31 | 67.15 | 72.08 | 0.468 |
|  | Kmer | 82.22 | 60.57 | 65.85 | 0.367 |
|  | Moran | 85.10 | 57.14 | 60.70 | 0.28 |
| LR | One-hot | 74.56 | 74.23 | 68.41 | 0.415 |
|  | NCP | 57.83 | 55.59 | 55.63 | 0.071 |
|  | DACC | 56.5 | 69.23 | 62.33 | 0.250 |
|  | PseDNC | 63.46 | 63.38 | 63.41 | 0.265 |
|  | PseKNC | 69.56 | 53.93 | 58.80 | 0.210 |
|  | PseEIIP | 68.33 | 53.72 | 56.09 | 0.162 |
|  | PCPseDNC | 64.86 | 61.53 | 62.87 | 0.258 |
|  | PCPseTNC | 58.43 | 63.05 | 60.97 | 0.214 |
|  | Kmer | 63.46 | 60.01 | 61.51 | 0.232 |
|  | Moran | 58.08 | 59.90 | 59.07 | 0.179 |
| KNN | One-hot | 80.46 | 77.32 | 76.23 | 0.575 |
|  | NCP | 62.24 | 62.21 | 62.13 | 0.192 |
|  | DACC | 55.48 | 63.41 | 59.89 | 0.192 |
|  | PseDNC | 67.7 | 70.89 | 69.37 | 0.386 |
|  | PseKNC | 75.46 | 64.07 | 69.10 | 0.393 |
|  | PseEIIP | 73.91 | 68.75 | 71.0 | 0.423 |
|  | PCPseDNC | 68.20 | 67.85 | 68.02 | 0.360 |
|  | PCPseTNC | 66.87 | 68.88 | 68.02 | 0.355 |
|  | Kmer | 71.11 | 72.32 | 71.23 | 0.425 |
|  | Moran | 59.19 | 61.53 | 60.43 | 0.20 |
| DTREE | One-hot | 73.57 | 75.52 | 71.39 | 0.477 |
|  | NCP | 56.61 | 58.76 | 57.63 | 0.103 |
|  | DACC | 57.06 | 67.02 | 62.05 | 0.24 |
|  | PseDNC | 64.48 | 68.27 | 66.39 | 0.327 |
|  | PseKNC | 68.57 | 60.30 | 64.22 | 0.289 |
|  | PseEIIP | 61.94 | 60.19 | 60.97 | 0.220 |
|  | PCPseDNC | 62.29 | 63.97 | 63.14 | 0.262 |
|  | PCPseTNC | 61.73 | 70.52 | 65.85 | 0.322 |
|  | Kmer | 60.86 | 61.08 | 60.97 | 0.219 |
|  | Moran | 56.01 | 58.76 | 57.45 | 0.147 |
| SVM | One-hot | 79.13 | 81.15 | 80.06 | 0.648 |
|  | NCP | 62.975 | 63.28 | 63.11 | 0.212 |
|  | DACC | 52.38 | 62.77 | 57.45 | 0.152 |
|  | PseDNC | 67.66 | 68.31 | 68.02 | 0.358 |
|  | PseKNC | 74.13 | 65.12 | 69.37 | 0.392 |
|  | PseEIIP | 74.01 | 72.73 | 73.12 | 0.463 |
|  | PCPseDNC | 68.94 | 66.34 | 67.47 | 0.350 |
|  | PCPseTNC | 73.80 | 76.15 | 75.06 | 0.498 |
|  | Kmer | 73.11 | 72.77 | 73.44 | 0.468 |
|  | Moran | 59.77 | 62.05 | 60.97 | 0.217 |
| MLP | One-hot | 74.54 | 78.84 | 76.81 | 0.584 |
|  | NCP | 59.07 | 60.68 | 59.89 | 0.147 |
|  | DACC | 57.45 | 67.02 | 62.33 | 0.245 |
|  | PseDNC | 63.42 | 65.97 | 64.76 | 0.293 |
|  | PseKNC | 67.93 | 61.08 | 64.44 | 0.290 |
|  | PseEIIP | 63.26 | 65.26 | 64.22 | 0.285 |
|  | PCPseDNC | 63.58 | 62.31 | 62.87 | 0.257 |
|  | PCPseTNC | 69.54 | 73.84 | 71.81 | 0.434 |
|  | Kmer | 65.93 | 65.77 | 65.85 | 0.317 |
|  | Moran | 61.45 | 64.21 | 62.87 | 0.256 |
| SVM BAGGING | One-hot | 79.13 | 83.37 | 80.06 | 0.65 |
|  | NCP | 63.48 | 64.93 | 64.23 | 0.233 |
|  | DACC | 61.44 | 68.47 | 65.31 | 0.299 |
|  | PseDNC | 68.8 | 71.95 | 70.46 | 0.408 |
|  | PseKNC | 74.13 | 65.12 | 69.37 | 0.392 |
|  | PseEIIP | 70.5 | 69.84 | 70.18 | 0.40 |
|  | PCPseDNC | 68.57 | 68.55 | 68.56 | 0.37 |
|  | PCPseTNC | 71.73 | 78.37 | 75.06 | 0.50 |
|  | Kmer | 70.15 | 71.91 | 71.02 | 0.42 |
|  | Moran | 62.56 | 65.26 | 63.95 | 0.278 |
| G-BOOSTING | One-hot | 75.16 | 75.35 | 73.56 | 0.51 |
|  | NCP | 57.87 | 59.48 | 58.74 | 0.120 |
|  | DACC | 54.09 | 63.97 | 59.07 | 0.18 |
|  | PseDNC | 64.5 | 67.01 | 65.85 | 0.31 |
|  | PseKNC | 70.16 | 62.76 | 66.38 | 0.33 |
|  | PseEIIP | 64.22 | 65.36 | 64.76 | 0.29 |
|  | PCPseDNC | 66.28 | 66.49 | 66.39 | 0.32 |
|  | PCPseTNC | 66.47 | 70.35 | 68.56 | 0.36 |
|  | Kmer | 63.14 | 62.46 | 63.14 | 0.26 |
|  | Moran | 54.14 | 57.44 | 55.82 | 0.11 |
| CNN | One-hot | 96.12 | 92.63 | 95.68 | 0.87 |
|  | NCP | 66.69 | 64.07 | 64.19 | 0.58 |
|  | DACC | 62.41 | 69.59 | 65.30 | 0.53 |
|  | PseDNC | 73.50 | 71.41 | 71.12 | 0.68 |
|  | PseKNC | 78.54 | 65.63 | 69.81 | 0.62 |
|  | PseEIIP | 72.94 | 68.91 | 69.56 | 0.64 |
|  | PCPseDNC | 72.78 | 68.43 | 69.33 | 0.63 |
|  | PCPseTNC | 73.63 | 74.70 | 73.55 | 0.71 |
|  | Kmer | 73.60 | 69.43 | 70.36 | 0.68 |
|  | Moran | 66.15 | 64.5 | 64.47 | 0.62 |

# Table s4. Second layer performance of baseline models on different feature encoding techniques

| **Classifier** | **Encoding Scheme** | **Sn** | **Sp** | **Acc** | **MCC** |
| --- | --- | --- | --- | --- | --- |
| AdB | One-hot | 94.5 | 94.9 | 91.2 | 0.75 |
|  | NCP | 71.43 | 85.27 | 81.98 | 0.46 |
|  | DACC | 40 | 81.0 | 72.23 | 0.16 |
|  | PseDNC | 80.1 | 98.8 | 96.2 | 0.87 |
|  | PseKNC | 96.1 | 99.9 | 95.2 | 0.85 |
|  | PseEIIP | 80.2 | 95.5 | 92.0 | 0.75 |
|  | PCPseDNC | 98.9 | 97.2 | 96.2 | 0.90 |
|  | PCPseTNC | 99.5 | 76.16 | 80.0 | 0.58 |
|  | Kmer | 66.66 | 90.99 | 88.0 | 0.51 |
|  | Moran | 50.0 | 82.6 | 76.0 | 0.28 |
| NB | One-hot | 79.9 | 79.4 | 80.2 | 0.56 |
|  | NCP | 63.55 | 71.75 | 72.29 | 0.29 |
|  | DACC | 30.13 | 84.36 | 80.2 | 0.19 |
|  | PseDNC | 78.23 | 84.4 | 85.2 | 0.62 |
|  | PseKNC | 70.23 | 80.1 | 80.5 | 0.71 |
|  | PseEIIP | 50.23 | 80.0 | 80.0 | 0.32 |
|  | PCPseDNC | 50.0 | 82.0 | 82.0 | 0.30 |
|  | PCPseTNC | 50.0 | 64.0 | 64.0 | 0.14 |
|  | Kmer | 50.0 | 84.0 | 84.0 | 0.15 |
|  | Moran | 26.23 | 82.60 | 76.23 | 0.12 |
| XG BOOST | One-hot | 94.9 | 86.3 | 87.2 | 0.58 |
|  | NCP | 77.86 | 79.53 | 80.15 | 0.34 |
|  | DACC | 98.99 | 87.5 | 88.8 | 0.46 |
|  | PseDNC | 99.9 | 87.5 | 88.0 | 0.47 |
|  | PseKNC | 66.6 | 86.3 | 84.23 | 0.43 |
|  | PseEIIP | 50.23 | 80.0 | 80.0 | 0.32 |
|  | PCPseDNC | 98.9 | 90.9 | 92.2 | 0.73 |
|  | PCPseTNC | 98.5 | 72.7 | 76.0 | 0.49 |
|  | Kmer | 99.5 | 91.30 | 92.0 | 0.67 |
|  | Moran | 50.23 | 80.0 | 80.0 | 0.32 |
| LR | One-hot | 77.2 | 81.95 | 83.8 | 0.57 |
|  | NCP | 51.10 | 76.59 | 75.18 | 0.19 |
|  | DACC | 33.33 | 86.36 | 80.2 | 0.17 |
|  | PseDNC | 82.2 | 84.4 | 83.2 | 0.72 |
|  | PseKNC | 78.23 | 80.23 | 79.2 | 0.68 |
|  | PseEIIP | 55.0 | 86.76 | 88.8 | 0.28 |
|  | PCPseDNC | 50.0 | 80.0 | 80.0 | 0.30 |
|  | PCPseTNC | 50.0 | 64.0 | 62.0 | 0.14 |
|  | Kmer | 50.0 | 84.0 | 84.0 | 0.15 |
|  | Moran | 50.0 | 86.95 | 84.0 | 0.27 |
| KNN | One-hot | 94.5 | 90.5 | 91.2 | 0.72 |
|  | NCP | 81.58 | 85.68 | 85.61 | 0.52 |
|  | DACC | 33.3 | 86.36 | 80.2 | 0.17 |
|  | PseDNC | 98.9 | 95.45 | 96.0 | 0.84 |
|  | PseKNC | 97.6 | 90.9 | 92.5 | 0.73 |
|  | PseEIIP | 98.9 | 90.9 | 92.0 | 0.73 |
|  | PCPseDNC | 98.9 | 95.5 | 96.0 | 0.87 |
|  | PCPseTNC | 98.9 | 80.0 | 84.0 | 0.66 |
|  | Kmer | 99.5 | 95.4 | 96.2 | 0.84 |
|  | Moran | 66.6 | 90.9 | 88.0 | 0.51 |
| DTREE | One-hot | 78.33 | 93.9 | 83.8 | 0.57 |
|  | NCP | 43.77 | 84.47 | 75.38 | 0.22 |
|  | DACC | 25.2 | 85.71 | 76 | 0.10 |
|  | PseDNC | 25.3 | 96.96 | 74.0 | 0.12 |
|  | PseKNC | 75.5 | 90.04 | 88.2 | 0.60 |
|  | PseEIIP | 60.0 | 90.0 | 84.0 | 0.50 |
|  | PCPseDNC | 62.5 | 98.9 | 88.8 | 0.72 |
|  | PCPseTNC | 83.33 | 78.94 | 80.0 | 0.55 |
|  | Kmer | 33.3 | 89.47 | 76.0 | 0.26 |
|  | Moran | 25.0 | 85.71 | 76.0 | 0.10 |
| SVM | One-hot | 94.9 | 93.9 | 91.23 | 0.69 |
|  | NCP | 73.70 | 84.27 | 83.60 | 0.44 |
|  | DACC | 32.2 | 83.3 | 80.2 | 0.18 |
|  | PseDNC | 98.9 | 95.45 | 96.23 | 0.84 |
|  | PseKNC | 97.6 | 90.9 | 92.2 | 0.73 |
|  | PseEIIP | 99.9 | 86.9 | 88.0 | 0.58 |
|  | PCPseDNC | 71.42 | 98.9 | 92.2 | 0.80 |
|  | PCPseTNC | 98.9 | 80.0 | 84.0 | 0.66 |
|  | Kmer | 98.5 | 95.4 | 96.0 | 0.84 |
|  | Moran | 32.2 | 83.3 | 80.0 | 0.08 |
| MLP | One-hot | 94.5 | 90.4 | 91 | 0.69 |
|  | NCP | 61.58 | 78.90 | 76.82 | 0.32 |
|  | DACC | 25.23 | 85.9 | 76.0 | 0.10 |
|  | PseDNC | 70.28 | 84.2 | 80.23 | 0.68 |
|  | PseKNC | 99.5 | 86.9 | 88.1 | 0.58 |
|  | PseEIIP | 50.5 | 80.0 | 80.0 | 0.30 |
|  | PCPseDNC | 72.0 | 84.2 | 82.23 | 0.62 |
|  | PCPseTNC | 50.0 | 64.0 | 64.0 | 0.14 |
|  | Kmer | 98.5 | 95.4 | 96.0 | 0.84 |
|  | Moran | 66.6 | 90.6 | 88.0 | 0.51 |
| SVM BAGGING | One-hot | 94.5 | 93.98 | 91 | 0.50 |
|  | NCP | 63.68 | 87.26 | 82.18 | 0.41 |
|  | DACC | 20.23 | 85.23 | 72.12 | 0.05 |
|  | PseDNC | 66.6 | 90.9 | 88.8 | 51.0 |
|  | PseKNC | 80.2 | 95.2 | 92.5 | 0.75 |
|  | PseEIIP | 98.9 | 95.25 | 96.0 | 0.87 |
|  | PCPseDNC | 99.5 | 95.23 | 96.0 | 0.87 |
|  | PCPseTNC | 84.0 | 98.98 | 84.0 | 0.64 |
|  | Kmer | 66.66 | 90.9 | 88.0 | 0.510 |
|  | Moran | 33.33 | 86.36 | 80.0 | 0.174 |
| G-BOOSTING | One-hot | 93.5 | 90.5 | 91 | 0.72 |
|  | NCP | 55.97 | 85.27 | 79.55 | 0.36 |
|  | DACC | 16.6 | 84.21 | 68.2 | 0.10 |
|  | PseDNC | 60.2 | 95.5 | 88.0 | 0.60 |
|  | PseKNC | 98.5 | 95.2 | 96.0 | 0.87 |
|  | PseEIIP | 75.0 | 90.47 | 88.0 | 0.60 |
|  | PCPseDNC | 57.14 | 94.44 | 84.0 | 0.57 |
|  | PCPseTNC | 87.5 | 88.8 | 88.0 | 0.73 |
|  | Kmer | 66.6 | 90.9 | 88.0 | 0.51 |
|  | Moran | 26.23 | 82.60 | 76.23 | 0.12 |
| CNN | One-hot | 94.32 | 98.5 | 97.25 | 0.86 |
|  | NCP | 69.42 | 86.90 | 84.27 | 0.50 |
|  | DACC | 45.498 | 88.687 | 81.615 | 0.51 |
|  | PseDNC | 81.061 | 96.356 | 92.586 | 0.13 |
|  | PseKNC | 91.006 | 94.567 | 93.863 | 0.56 |
|  | PseEIIP | 76.886 | 92.578 | 91.88 | 0.55 |
|  | PCPseDNC | 80.926 | 96.727 | 93.963 | 0.56 |
|  | PCPseTNC | 85.063 | 81.758 | 81.6 | 0.54 |
|  | Kmer | 81.412 | 94.966 | 93.14 | 0.55 |
|  | Moran | 53.509 | 89.532 | 85.543 | 0.29 |
